# Entrained Flow Gasification of Polypropylene Pyrolysis Oil

**DOI:** 10.3390/molecules26237317

**Published:** 2021-12-02

**Authors:** Fredrik Weiland, Muhammad Saad Qureshi, Jonas Wennebro, Christian Lindfors, Taina Ohra-aho, Hoda Shafaghat, Ann-Christine Johansson

**Affiliations:** 1 RISE Energy Technology Center AB, Box 726, SE-941 28 Piteå, Sweden; jonas.wennebro@ri.se (J.W.); hoda.shafaghat@ri.se (H.S.); ann-christine.johansson@ri.se (A.-C.J.); 2 VTT Technical Research Centre of Finland Ltd., P.O. Box 1000, FI-02044 VTT Espoo, Finland; muhammad.qureshi@vtt.fi (M.S.Q.); christian.lindfors@vtt.fi (C.L.); taina.ohra-aho@vtt.fi (T.O.-a.)

**Keywords:** pyrolysis, gasification, syngas, chemical recycling, plastic waste

## Abstract

Petrochemical products could be produced from circular feedstock, such as waste plastics. Most plants that utilize syngas in their production are today equipped with entrained flow gasifiers, as this type of gasifier generates the highest syngas quality. However, feeding of circular feedstocks to an entrained flow gasifier can be problematic. Therefore, in this work, a two-step process was studied, in which polypropylene was pre-treated by pyrolysis to produce a liquid intermediate that was easily fed to the gasifier. The products from both pyrolysis and gasification were thoroughly characterized. Moreover, the product yields from the individual steps, as well as from the entire process chain, are reported. It was estimated that the yields of CO and H_2_ from the two-step process were at least 0.95 and 0.06 kg per kg of polypropylene, respectively, assuming that the pyrolysis liquid and wax can be combined as feedstock to an entrained flow gasifier. On an energy basis, the energy content of CO and H_2_ in the produced syngas corresponded to approximately 40% of the energy content of the polypropylene raw material. This is, however, expected to be significantly improved on a larger scale where losses are proportionally smaller.

## 1. Introduction

The global production of plastics reached almost 360 million tons in 2018, whereof approximately 62 million tons was produced in Europe [1]. Polyolefins, such as polyethylene (PE) and polypropylene (PP), account for a large part of the market. These plastics are widely used for food packaging, bags, plastic films, toys, pipes, or in automotive parts. Polypropylene accounted for more than 19% of the European demand in 2018 [1]. The enormous use of plastics has some negative consequences globally, as plastic pollution is widespread worldwide and problems caused by plastic wastes are one of the major challenges nowadays [2]. For instance, the pollution of microplastics in the marine environment is severe [3], which endangers both ecosystems and human health [4]. Therefore, several countries are introducing policies and legislation aiming to reduce the use of fossil plastic materials to tackle the related challenges [5]. However, plastic materials are often advantageous over the alternatives [6]. For example, in the packaging industry, plastics are often cheaper than the green alternatives produced from, e.g., paper, and the plastic materials are superior to prolong the life of food in both supermarkets and homes [5]. Due to the lightweight nature of plastic packaging materials, the environmental cost is usually much lower compared to the alternatives. Glass bottles and glass jars in the food chain, for example, were found to result in a higher environmental burden than plastics [7,8,9,10]. Thus, plastic itself is not the problem, but the way of plastic waste management is important [11]. The European strategy for plastics is, therefore, to create a sustainable plastics industry by improving recycling and thereby reduce the climate impact. This includes, among other things, improving both the waste collection system and the economics of plastic recycling by better product design, making the recycling easier [12].

Mechanical recycling is probably the most straightforward method for plastic waste management. However, this recycling strategy is limited due to the requirement of rather pure polymer streams. Without proper sorting, plastics will be downcycled into lower value plastic materials [13]. Even with proper handling, pure polymers degrade after several cycles of mechanical recycling [14,15,16]. Eriksen et al. concluded that, especially, mixed polypropylene (PP) waste could not facilitate closed-loop recycling into new products due to the limitations caused by polymer degradation during the mechanical recycling process [14]. The natural degradation of plastic during its life caused by its age and use also limits the applicability of the material for closed loop recycling.

Chemical recycling may be an alternative to such plastics that are not suitable for mechanical recycling. Chemical recycling usually involves thermochemical processes that break down the polymers into smaller molecules such as gases and/or liquids [17]. These intermediate products can subsequently be used as feedstocks in the petrochemical industry to produce new chemicals and/or plastic raw materials [18]. Chemical recycling is thus an opportunity to regain the value of the plastic after many cycles of mechanical recycling, or to be used when mechanical recycling is not possible, e.g., for mixed and heterogeneous plastic wastes. Pyrolysis and gasification are two thermochemical processes with high technology readiness level (TRL) that can be used to process mixed plastic wastes [19].

Pyrolysis is a thermochemical process in which the feedstock is decomposed to smaller molecules using heat under an inert atmosphere. One of the pioneering studies on the pyrolysis of plastic waste and its potential was made by Scott et al. [20]. The product obtained from pyrolysis relies on several factors ranging from the type of plastic [21], reactor used [22], operating conditions (temperature, pressure, residence time, and heating rate), and the use of a catalyst [23]. The influence of these operational parameters is assessed in several articles [22,24,25,26,27,28,29]. The hydrocarbon range obtained is significantly altered by the variation of these influencing parameters. Fast pyrolysis of plastic waste is normally carried out in fluidized bed reactor and the residence time is nearly 1 s. The primary product obtained by fast pyrolysis of plastics at moderate temperatures (around 500 °C) is wax, which contains molecules with a carbon number above C_20_ [30]. The influence of residence time and temperature is significant [29,31,32] in the modification of the pyrolysis yields and product selectivity. Depending on the feedstock and the pyrolysis process conditions, the yields of gas, condensables (pyrolysis oil and wax), and solid (char) products differ [33]. Moderate temperatures (around 500 °C), fast heating rates and short vapor residence times generally result in a greater yield of pyrolysis oil/wax than gases and char. On the contrary, low temperatures, slow heating rates and long vapor residence time generally result in an improved yield of char at the expense of pyrolysis oil yield [34]. The liquid product from pyrolysis of plastics typically contains heavy oil, light oil, mid-distillates, and naphtha [35]. Depending on the feedstock, the heavier oils contain paraffins, olefins, aromatics, and high molecular weight components, typically with boiling points greater than 250 °C. This wax-like fraction may appear solid at room temperature. Pyrolysis of polyethylene (PE), for instance, generated a relatively large proportion of wax at low pyrolysis temperature (450 °C) [21]. On the other hand, a higher temperature (above 600 °C) resulted in wax breaking down into shorter components, together with an enhanced gas formation [33]. The liquid product from waste plastics pyrolysis can potentially be utilized as a feedstock in the petrochemical industry, where it for example replaces fossil naphtha to produce olefins via the steam cracking process [36].

As mentioned above, gasification is another high-TRL thermochemical conversion technology beside pyrolysis that could be used for waste plastics in the petrochemical industry. Lopez et al. [37] compiled a comprehensive overview of the most common gasification technologies for waste plastics, including their general advantages and disadvantages. Additionally, Ciuffi et al. [38] reviewed supercritical water gasification of plastic waste in the context of other available gasification technologies. In summary, both studies concluded that different types of fluidized bed gasifiers were widely described in the plastic waste gasification literature alongside fixed-beds, spouted beds, and plasma reactors. The major challenge reported for gasification of plastic wastes was tar formation, which can be severe from low-temperature gasification processes, such as fluidized beds that are limited in temperature due to the risk of bed agglomeration ([37,38] and references therein). Supercritical water gasification has the advantage of reduced tar formation, resulting in a cleaner syngas. However, the technology is still poorly studied. Process complexity and high costs are reasons that make large-scale implementation difficult [38].

The petrochemical industry currently produces syngas mainly through the conversion of fossil raw materials. When it comes to production of high-quality syngas for downstream synthesis of fuels and chemicals, the entrained flow gasification technology is the preferred option. Entrained flow gasifiers therefore account for most installations around the world [39]. Essentially all commercial entrained flow gasifiers are of the autothermal type, meaning that part of the feedstock is consumed to provide the necessary heat for the process. Thus, the process temperature in these high-temperature reactors is mainly controlled by the oxygen equivalence ratio, λ, which also affects the overall efficiency of the gasification process [39,40]. A too high λ reduces the chemical energy in the syngas due to promotion of combustion reactions converting the desired energetic gases, carbon monoxide (CO), and hydrogen (H_2_), to carbon dioxide (CO_2_) and water vapor (H_2_O). On the other hand, reducing λ too far results in a too low gasification temperature where complete fuel conversion cannot be obtained. All in all, this poses a challenge in terms of optimizing the operation of the gasifier. Thus, it is important to have a sufficiently high process temperature to convert unwanted hydrocarbons to syngas, and to minimize tar and soot formation, without at the same time sacrificing too much of the overall gasification efficiency for downstream synthesis [41].

The high temperatures used in entrained flow gasifiers (approximately 1200–1500 °C) minimize the need for downstream syngas tar cleaning compared to other gasification processes that operate at lower temperatures. However, the residence time inside an entrained flow gasifier is short, in the order of a few seconds. Therefore, the feedstock must be dispersed into small particles or droplets to ensure full conversion inside the reactor. One problem with plastic waste is that it is difficult, almost impossible, to mill to fine powders. The plastics can melt and clog milling machines, or the generated powder becomes very lightweight, making it impossible to feed continuously into any gasifier. One alternative to overcome these problems is to pyrolyze the plastic waste, producing a liquid product that can be fed by ordinary pumps to entrained flow gasifiers with spray nozzle burners within the petrochemical industry.

Such a concept has already been developed by the Karlsruhe Institute of Technology (KIT), Germany, for the production of synthetic fuels from biomass residues [42]. The concept, bioliq^®^, is based on decentralized pyrolysis of dispersed biomass to produce a bioslurry with high energy density, which is thereafter fed to a centralized entrained flow gasification plant with downstream synthesis steps [43]. Plastic wastes are, similar to biomass residues, usually widely distributed and, due to its low energy density, the transportation becomes expensive. Production of high-quality chemicals/fuels from waste feedstocks requires usually complex plants, which scale is usually large due to economic reasons. A solution for this is to convert the waste plastic into liquid in decentralized pyrolysis plants and upgrade the oil in a centralized facility, as in the bioliq^®^ concept. Experimental investigations related to entrained flow gasification of pyrolysis oil from plastic waste are, however, scarce. Studies in the current scientific literature are usually about gasification of bio-based pyrolysis oil. For example, from straw- and wood derived pyrolysis oil [43,44], or the co-gasification of biomass derived pyrolysis oil together with Kraft black liquor from the pulp and paper industry [45,46,47].

The aim of this work was therefore to experimentally investigate the possibilities for chemical recycling of plastics via a two-step approach combining fluidized bed pyrolysis and entrained flow gasification processes. This concept may provide an opportunity for the petrochemical industry to replace fossil feedstocks with circular raw materials in the future. Polypropylene (PP), being one of the most produced plastics worldwide [1], was therefore chosen as the model compound. In the long term, the concept should of course be used for the most complex waste streams, while pure plastic streams such as polypropylene are better suited for other purposes. Nevertheless, this work resulted in initial data on expected yields from pyrolysis and gasification, as well as from the entire process chain. In addition, measured syngas compositions at different operating conditions during gasification are reported together with estimated process efficiency from polypropylene to syngas.

## 2. Results and Discussion

### 2.1. Pyrolysis

The product obtained from the pyrolysis of polypropylene was a mixture of liquid, wax (solid at room temperature), and gases. Fast pyrolysis of polypropylene resulted mainly in a waxy product, 53 wt.% (Table 1), which built up in the lines and on the walls of the condensation system, and eventually resulted in a reactor pressure increase. The waxy product was mechanically removed from the walls of the condensers after the experiment. The main liquid fraction was condensed in the water cooler, whereas the main part of wax was collected from the electric filters. Gas yield was still quite low, which is typical for fast pyrolysis of polyolefins at moderate conditions. Main gas components were propene and other light olefins, which could be separated for new polymer production or alternatively combusted to generate the heat for the pyrolysis process. Char or coke yield was negligible due to the clean feedstock used in the experiment. The product obtained from pyrolysis of polyproplyene in this study is in agreement with results from other acknowledged authors. Predel and Kaminsky [48] observed approximately 55 wt% of light and heavy waxes and 7 wt% gases during the pyrolysis of polypropylene in a fluidized bed reactor operating at approximately 515 °C. The product obtained from the pyrolysis of polymers are dependent on many factors, including the reactor type. Westerhout et al. [49] studied the influence of reaction conditions in the pyrolysis of polypropylene in a tubular reactor. Reactor temperature was varied from 650 °C to 800 °C and the residence time was varied from 0.1 to 1 s. An increase in the temperature led to the increase in the formation of ethane. The higher temperature combined with relatively longer residence time (1 s) led to the formation of butadiene, a ternary reaction product of ethane degradation.

The total yields are presented in Table 1. They did not sum up to 100 wt.% and around 16 wt.% were missing. It is unclear what the remaining product was, but most probably part of it was wax, which remained on the wall of the condensation system. The remainder was most likely light hydrocarbons (C_5+_) not captured in the liquid recovery system, nor measured by the gas analyses.

#### 2.1.1. Non-Condensable Gases

The yields of individual gas components including C_1_ to C_5_ n-alkanes and alkenes are presented in Table 2. In addition, gas compounds having carbon atoms from C_4_ to C_7_ were detected and quantified, but their structure was not measured in detailed. Propene was the major gas component formed during pyrolysis. This was in line with previously published results [49].

Other C_3+_ compounds include non-calibrated C_4_, C_5_, C_6_, and C_7_ hydrocarbons, with yields corresponding to 0.8, 0.4, 1.4, and 0.2 wt.%, respectively.

#### 2.1.2. Liquid and Waxy Products

After the experiment, waxes and liquids were separately characterized for physicochemical properties (Table 3). The elemental compositions (C, H, and N) were similar for the liquid and the wax, as well their heating values. The melting temperature of the wax was 98 °C, which was similar to previously reported values elsewhere [50]. The liquid product consisted mainly of lighter hydrocarbons (< C_20_), but also some light (Bp 340–500 °C) and heavy wax (Bp > 500 °C) components were present. This product fraction appeared as liquid at room temperature, whereas the waxy product was not. The wax fraction was instead enriched in hydrocarbons larger than C_20, i.e._, both lighter (C_21_–C_37_) and heavier wax components (up to C_102_) were present.

**Figure 1:**
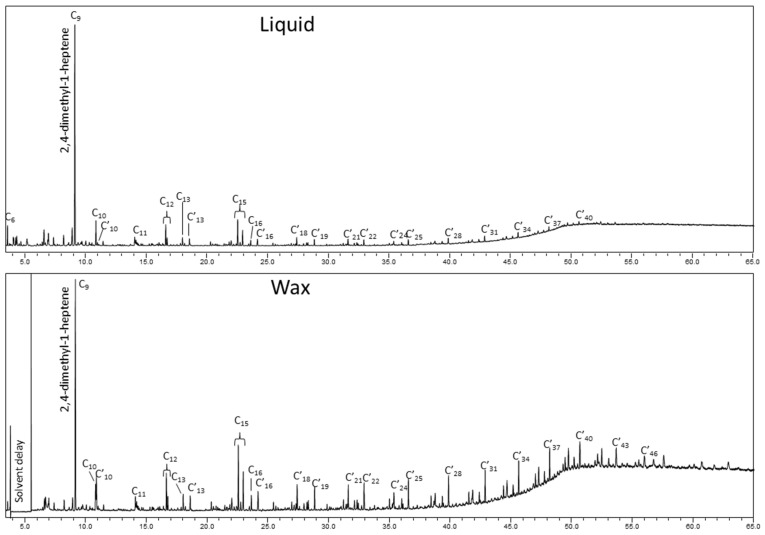
Total ion gas chromatograms of liquid (above) and wax (below) from PP pyrolysis at 600 °C. Peak identifications: C_6_ 2-methyl-1-pentene; C_9_ 2,4-dimethyl-1-heptene; C_10_ 2,4,6-trimethyl-1-heptene; C’_10_ 2,4,6-trimethyl-1,6-heptadiene; C_11_ 4,6-dimethyl-2-nonene (meso form); C_12_ 2,4,6-trimethyl-1-nonene (meso form) and 2,4,6-trimethyl -1-nonene (racemic form); C_13_ 2,4,6,8-tetramethyl-1-nonene (meso form); C’_13_ 2,4,6,8-tetramethyl-1,8-nonadiene (meso form); C_15_ 2,4,6,8-tetramethyl-1-undecene (isotactic), 2,4,6,8- tetramethyl-1-undecene (heterotactic) and 2,4,6,8-tetramethyl-1-undecene (syndiotactic); C_16_ 2,4,6,8,10-pentamethyl-1-undecene (isotactic); C’_16_ 2,4,6,8,10-pentamethyl-1,10-undecadiene (isotactic); C_18_ 2,4,6,8,10-pentamethyl-1-tridecene (isotactic); C’_19_ 2,4,6,8,10,12-hexamethyl-1,12-tridecadiene (isotactic); hydrocarbons series from i-C’_21_ to i-C’_46_.

The composition of liquid and wax were analyzed by GC/MS in more detail. The polypropylene feedstock had a branched structure and was, therefore, converted into branched hydrocarbons such as dienes, alkanes, and alkenes with increasing hydrocarbon number. The identities of the main compounds are presented in Figure 1. The major compound found in both fractions was 2,4-dimethyl-1-heptene (C_9_). This observation is similar to what has been reported elsewhere at high temperature pyrolysis of polypropylene [48]. The compounds were not quantified, but ratio between 2,4-dimethyl-1-heptene (C_9_) and hydrocarbons from C_6_ up to C_46_ was calculated being 0.4 and 0.2 for liquid and wax, respectively. The liquid fraction was selected for the subsequent gasification experiments described below. This allowed fuel feeding at room temperature.

### 2.2. Gasification

#### 2.2.1. Syngas Yields and Elemental Mass Balances

**Figure 2:**
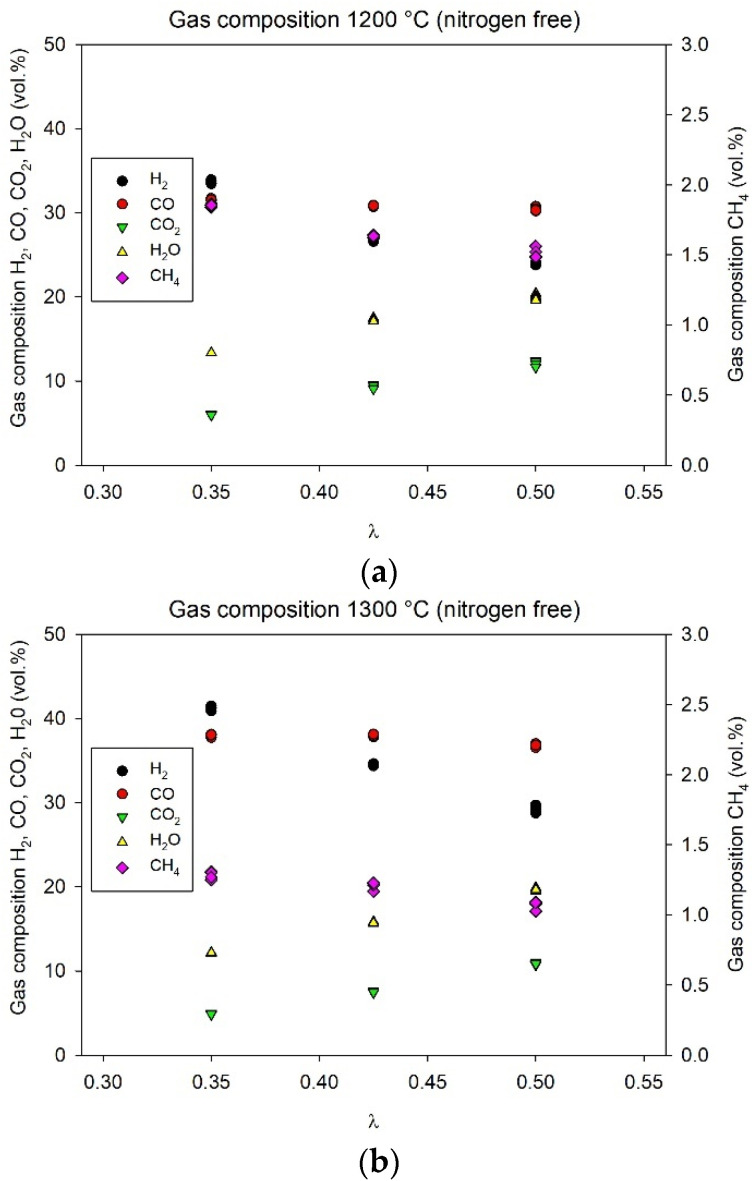
Main gas composition (vol.%) from gasification at (**a**) 1200 °C and (**b**) 1300 °C. Note that the composition is presented as nitrogen free.

Gasification of the liquid product from pyrolysis of polypropylene resulted in the main gas composition summarized in Figure 2. The experimental results are also presented as yields (mol/kg fuel) in Table 4.

From the experiments, the highest yield of CO was 51.2 mol/kg fuel (at λ = 0.43, 1300 °C) and the highest yield of H_2_ was 52.4 mol/kg fuel (at λ = 0.35, 1300 °C). For both tested temperatures, an increase in λ reduced the yield of H_2_, as expected, due to the combustion reactions forming H_2_O when more oxygen was available for reaction. Similarly, more CO_2_ was produced, while the yields of C_2_H_2_ and C_2_H_4_ decreased with increased λ setpoint for both temperatures. Increasing the gasification temperature increased the yields of H_2_ and CO, while reducing the yields of H_2_O, CO_2_, CH_4_, C_2_H_2_, and C_2_H_4_. This was consistent with other work on entrained flow gasification (e.g., [51,52]).

However, the yield of CH_4_ was not significantly affected by the λ setpoint (Table 4). This was expected, as previous experience showed that the CH_4_ yield was far from theoretical equilibrium and seemed to be mainly controlled by the gasification temperature [40]. Other studies showed that the gas phase conversion of CH_4_ was kinetically limited at relevant gasification temperatures [53,54,55]. The higher gasification temperature was therefore considered to improve the kinetics, which in turn resulted in CH_4_ concentration closer to the theoretical equilibrium.

Surprisingly, the yield of CO did not decrease when more oxygen was added to the gasification process. The CO yield was rather constant over the tested λ range at 1300 °C, and even increased with λ during gasification at 1200 °C. Billaud et al. [51] previously reported similar behaviour from entrained flow gasification of biomass in a drop tube furnace operated at different λ and temperatures. In this work, the increase in CO yield was most likely caused by an increased carbon conversion and a lower production of soot at higher λ settings or higher gasification temperature. Estimated mass balances for carbon, hydrogen, and oxygen are presented in Table 5. The mass balances were based on the yields measured by the gas analysis instruments at the exit of the gasifier and the corresponding feed rates of the elements at the gasifier inlet.

The table shows that approximately 80 wt.% of the feedstock carbon was found in the syngas (as CO, CO_2_, CH_4_, C_2_H_2_, and C_2_H_4_) during gasification at λ = 0.35 and 1200 °C. This number increased to 96 wt.% when λ was increased to 0.50 at 1200 °C. At the higher gasification temperature, 1300 °C, there was a similar trend, but here the lowest and highest values were 86 wt.% and 99 wt.%, respectively. Thus, the results indicate that the carbon conversion to syngas increased with both higher λ and gasification temperature. This was consistent with the results found by other researchers. For example, Qin et al. [52], studied biomass gasification at different temperatures (1000–1350 °C) and λ (0.25–0.50) in a similar reactor that was used in this study. It was concluded that the gasification temperature affected the reactions that control tar and soot formation and destruction. At low temperature (1000 °C), Qin and co-workers reported high tar yield. Increasing the gasification temperature resulted in the conversion of tar species, but at the expense of increased soot formation up to a certain point. A maximum soot yield was obtained at 1200 °C, whereas the soot yield was reduced to approximately 60% of the maximum value at 1350 °C. Moreover, at a gasification temperature of 1350 °C, the soot yield at λ = 0.50 was approximately 40% of the soot yield found at λ = 0.35. Almost no tar was measured at the higher operating temperatures [52]. Based on the study by Qin et al., it is assumed that tar production was minimal at the relatively high gasification temperatures used in this study.

Billaud et al. [51] concluded that soot was not consumed directly by the added oxygen at higher λ. Instead, they claimed that soot formation was inhibited by the OH radicals that reacted with the soot precursors, which resulted in reduced levels of soot at higher λ.

As previously mentioned, commercial scale entrained flow gasifiers are normally autothermal, meaning that λ and process temperature are interconnected such that λ in practice controls the process temperature (e.g., [39,40]). Additionally, from previous works with autothermal gasification of biomass, it has been shown that soot production was high by operating the entrained flow gasifier at λ setpoints, resulting in process temperatures around 1200 °C [56,57]. For example, the particulate matter in syngas produced from stem wood sawdust at different operating conditions was sampled by an online soot particle aerosol mass spectrometer (SP-AMS, from Aerodyne Inc., Billerica, MA, USA). The pressurized entrained flow biomass gasifier (PEBG) was operated at three different λ setpoints, 0.50, 0.45, and 0.40, resulting in corresponding process temperatures of approximately 1340 °C, 1290 °C, and 1230 °C, respectively. It was found that the soot mass concentration was almost four times higher at 1230 °C compared to the soot level measured at 1340 °C [56]. Another work [57] measured the soot particle concentration using a low-pressure impactor (from Dekati Ltd., Kangasala, Finland) sampling from a probe installed in the hot part of the PEBG. The results showed that the soot yield was more than three times higher at 1200 °C compared to the setpoint at 1400 °C [57].

#### 2.2.2. Gasification Performance Parameters

**Figure 3:**
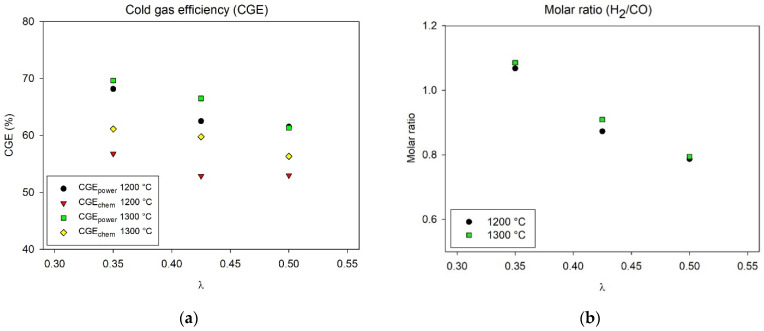
(**a**) Cold gas efficiency (CGE), and (**b**) Molar ratio (H_2_/CO), at different operating conditions during gasification.

The cold gas efficiencies (CGE_power_ and CGE_chem_) and the molar ratio of H_2_/CO for all experimental setpoints are presented in Figure 3. Both CGEs were reduced with an increase in λ-setpoint. Increasing the process temperature resulted especially in an improvement of the CGE_chem_. The maximum CGE_power_ was about 70% and the maximum CGE_chem_ was about 62% (both obtained at 1300 °C, λ = 0.35). These numbers are in line with what can be expected, and similar to previously reported values from entrained flow gasification of biomass [40,58]. The molar ratio of H_2_/CO varied between about 0.8 to 1.1, depending on operating condition (λ). Increasing λ reduced the H_2_/CO ratio, primarily due to the consumption of H_2_. The process temperature was found not to affect the ratio significantly.

From a practical perspective concerning operation of commercial scale gasification plants, there will thus be a balance between obtaining adequate fuel conversion with minimal soot production, and at the same time avoiding burning too much of the energetic components in the syngas.

#### 2.2.3. Filter Deposits

**Figure 4:**
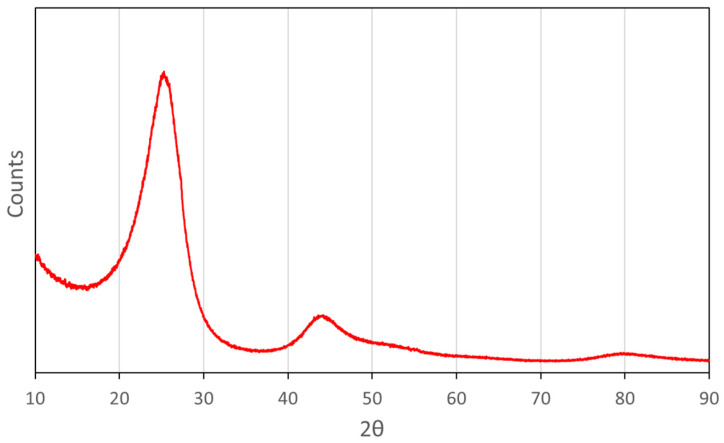
Results from XRD-analysis on the deposits on the heated filter after gasification at 1200 °C.

The deposits on the heated filter were collected and analysed with SEM/EDS and XRD. The SEM/EDS results showed that the deposits were composed of about 95 mol.% carbon and 4 mol.% oxygen. The XRD-pattern (Figure 4) showed two broad peaks at diffraction angles (2θ) corresponding approximately to 25° and 44°, respectively, and a much weaker peak near 80°. These diffraction maxima were associated with the (002), (100), and (110) planes, respectively, which are commonly found in disordered soot produced from hydrocarbon combustion [59]. Thus, the SEM and XRD analyses together indicated that the filter deposits were mainly composed of soot with a turbostratic structure.

### 2.3. Overall Process Performance

The results of the experiments indicated that the yield of liquid and waxy material from the pyrolysis step corresponded to at least 66 wt.% on this relatively small scale. As pyrolysis liquid and wax had similar elemental composition and calorific value (Table 3), both fractions were assumed to give similar results in high-temperature gasification. On a larger scale, feeding of waxy material could be made possible by heating until an adequate viscosity of the feedstock is reached. This ensures pumping and efficient atomization of the fuel into the gasification reactor.

Under the assumption that it is possible to combine the liquid and wax fractions, and from the experimental yields at λ = 0.43 and 1300 °C, it was estimated that at least 0.95 kg CO and 0.06 kg H_2_, respectively, can be produced per kg of polypropylene. Thus, a carbon efficiency corresponding to 48 wt.% from polypropylene to CO. The remaining carbon atoms were lost to pyrolytic gases (17 wt.%) and to the missing fraction of wax and/or undetected hydrocarbons from pyrolysis (16 wt.%). In addition, the gasification step resulted in carbon atoms from the polypropylene raw material being mainly distributed to CO_2_ (9 wt.%), CH_4_ (1 wt.%), C_2_H_2_ (1 wt.%), and soot (6 wt.%).

On an energy basis, 66% of the polypropylene heating value (LHV) ended up in the liquid and wax fractions after pyrolysis. Furthermore, the gasification setpoint of λ = 0.43 and 1300 °C resulted in a CGE_chem_ corresponding to 60%. This indicates an overall energy efficiency for the two-step approach from polypropylene to syngas (CO + H_2_) corresponding to about 40%. This is expected to be significantly improved on a large scale, where the recovery of waxy material from pyrolysis can be optimized, and the gasification conditions enhanced to minimize soot formation.

## 3. Materials and Methods

Pure polypropylene (PP) granules of particle size below 4 mm from Total (PPR 9220), Paris, France, was used as a model feedstock to demonstrate the value chain from waste plastics to syngas. The elemental composition of the polypropylene used in the experiment was measured to 86.4 wt.% C and 14.8 wt.% H, respectively. Pyrolysis of the polypropylene feedstock, as well as characterization of the pyrolysis products, were performed at VTT Technical Research Centre of Finland. The liquid product from pyrolysis was thereafter gasified in an entrained flow reactor at RISE Research Institute of Sweden.

### 3.1. Pyrolysis

**Figure 5:**
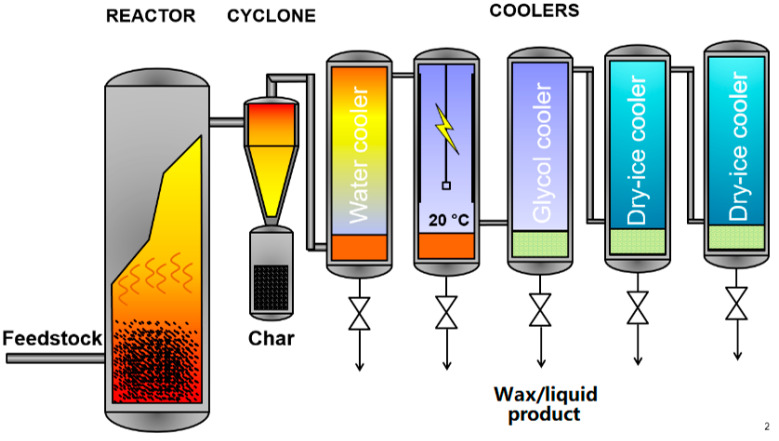
Bench scale unit used in the pyrolysis experiments.

The pyrolysis experiment was carried out in a bench scale fluidized bed reactor using nitrogen as fluidization medium (Figure 5). The pyrolysis temperature was 600 °C and vapor phase residence time 2 s. Approximately 300 g of quartz sand was used as an inert bed material in the experiment, and the feed rate of the polypropylene feedstock was close to 600 g/h. Two cyclones were used for the collection of solids after pyrolysis. Thereafter, hot vapours and gases were cooled rapidly using four coolers and one electrostatic precipitator. In the coolers, the temperature of the vapours was gradually decreased using water (40 °C), glycol (0 °C), and dry ice (−78.5 °C) as cooling media. Liquid and waxy product was collected from the liquid tap under each cooler after the experiment. All liquids, and all waxy products were combined into two separate samples before analyses. Gas bag samples were collected from the non-condensable gas and its composition was analysed by gas chromatography (GC) after the experiment. Liquid, wax, and char were weighed, and gas content analysed to establish a mass balance.

### 3.2. Analytical Methods

Elemental composition analysis (CHN) was carried out using an Elemental VARIOMAX CHN analyser (Staufen, Germany) and higher heating value (HHV) was determined using an IKA Werke C 5000 Control calorimeter (Hanau, Germany) according to standards ASTM D 5291 and DIN 51900, respectively. Product gas composition (H_2_, O_2_, N_2_, CO, CO_2_, CH_4_, and C_2_-C_4_) was analysed after the experiments with a micro-GC (Agilent 490-series, Middleburg, Netherlands). The gas chromatograph was equipped with a thermal conductivity detector (TCD). The composition of the liquid and wax products was analysed using a Shimadzu GCMS-QP2010 Ultra (Canby, Oregon, USA) gas chromatograph equipped with a mass selective quadrupole detector (GC-MSD). Separation of compounds was performed with a HP Ultra 1 (Agilent, Middleburg, Netherlands) fused silica capillary column (length: 50 m, inner diameter: 0.32 mm, and film thickness: 0.52 μm). Before analyses, liquids were dissolved in methylene chloride (emsure, Merck, Darmstadt, Germany) and waxes in tetrahydrofuran (emsure, Merck, Darmstadt, Germany) due to the partial insolubility in dichloromethane. Heavy hydrocarbons ranging from C_7_ to C_110_ were analysed with an Shumadzu Nexis GC -2030 (Darmstadt, Germany) with an on-column inlet and an Agilent (Middleburg, Netherlands) CP-SimDist UltiMetal high temperature metal-capillary column (10 m × 0.53 mm, film 0.17 µm, and 1 m retention gap) after dissolution of sample into carbon bisulphide (purity of 99.9%, Acros organics, Fair Lawn, UK). Melting temperature of the wax was visually measured by heating the sample under microscope. The temperature at which the sample started to change into liquid was recorded as the melting temperature.

### 3.3. Gasification

Gasification experiments were performed in an entrained flow drop tube furnace (DTF) using the liquid product from polypropylene pyrolysis as a feedstock, also referred to as “fuel”. The DTF consisted of a vertically mounted, 2100 mm long ceramic tube (Al_2_O_3_) with an inner diameter of 50 mm. The ceramic tube was placed inside an oven with 5 heating zones and sealed with water cooled endcaps at the top and bottom. The temperature and atmosphere within the DTF were controlled during the experiments. In addition, the experimental setup consisted of an injection system for fuel and gases, a particle collection system, and gas analysing instruments.

The fuel injection system was mounted onto the top endcap and consisted of a syringe pump mounted to a water-cooled fuel feeding probe. The fuel feeder dispensed the liquid feedstock through a capillary tube (OD: 1.6, ID: 0.5 mm) and the end of the capillary was welded onto a stainless-steel atomizing nozzle that was inserted at the tip of the liquid injection probe. The atomizer was based on a design by [60]. The same feeding system has previously been used by [61], where a more detailed description can be found. During operation two different gas flows were added to the injection system through mass flows controllers (MFC). The primary gas flow (air) was added trough the atomizing nozzle to form fine droplets of the oil. The secondary gas flow (nitrogen) was directed through a porous stainless-steel disc (20 µm pore size) placed vertically around the liquid feeder resulting in an entrained flow of gas downward through the ceramic tube.

**Figure 6:**
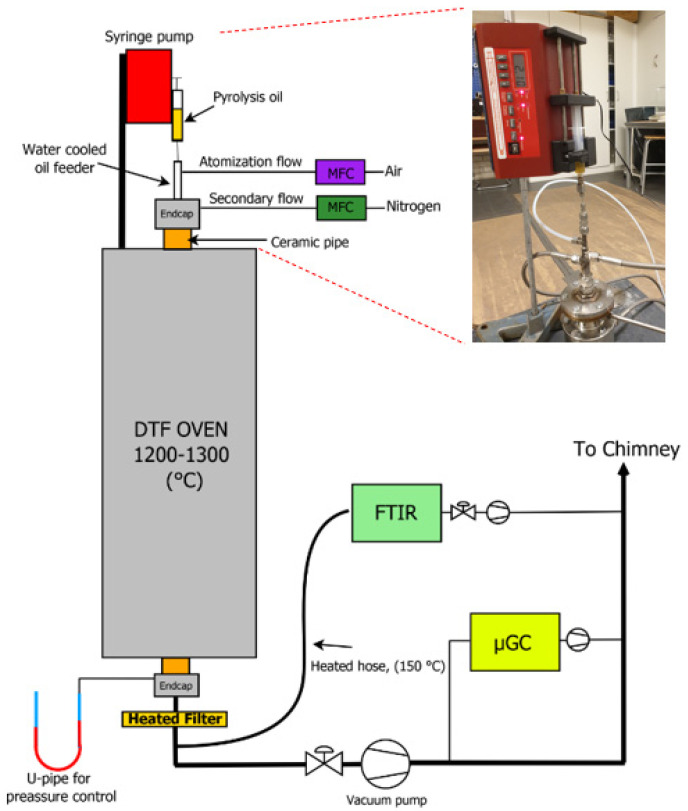
Schematics of the experimental setup used for gasification experiments in the DTF.

Directly after the DTF oven, the particle collection system was mounted onto the bottom endcap. Figure 6 shows a schematic process flow diagram of the gasification experimental setup. The particle sampling system consisted of a heated filter (200 °C) with a filter diameter of 90 mm. The filter (quartz microfibre) was used to capture any potential soot particles remaining after gasification. After the experiment, particulate material was analysed using scanning electron microscopy (SEM, Hitachi TM3030Plus, Tokyo, Japan) with energy dispersive X-ray spectroscopy (EDS) for elemental composition, and X-ray diffraction (XRD, Bruker D2 Phaser, Karlsruhe, Germany) for crystallinity.

After the filter, a partial syngas flow (1–2 L/min) was pumped through a Fourier transform infrared spectroscopy instrument (FTIR, MKS, Andover, MA, USA) that continuously analysed CO_2_, CO, H_2_O, CH_4_, and SO_2_. The pressure inside the DTF was controlled by regulating the gas flow to the vacuum pump at the gas exhaust (Figure 6). A micro gas chromatograph (µGC, Varian 490 GC, Middleburg, The Netherlands) equipped with two thermal conductivity detectors (TCD) continuously analysed the content of H_2_, N_2_, O_2_, CH_4_, CO, CO_2_, C_2_H_2_, C_2_H_4_, and C_2_H_6_ in the off gases.

Prior to the gasification experiments the liquid feedstock was centrifuged in a Sigma 2-16KL centrifuge (Sigma, Osterode am Harz, Germany) for 15 min at 5000 rpm. This was performed only to avoid any risk of plugging of the fine tubes of the injection system and atomization nozzle. Thus, a small fraction of bottom sediment was separated from the oil. This was not considered to affect the overall results or conclusions from the work.

Two process parameters, the furnace temperature (°C) and the stoichiometric oxygen equivalence ratio (λ), were varied during the gasification experiments. The λ was here defined as the ratio between the added air, ${\dot{m}}_{air}\;$(kg/s) and the stoichiometric air requirement for complete combustion, ${\dot{m}}_{air,\;stoich}\;$(kg/s), according to Equation (1).

(1) $$\lambda =\frac{{\dot{m}}_{air}}{{\dot{m}}_{air,stoich}}$$

The DTF is, in principle, an allothermal entrained flow gasifier, where the heat for gasification is supplied by the electrical heating element of the DTF oven. Thus, the gasification temperature was controlled by the DTF temperature setpoint, meaning that gasification temperature and λ could be controlled independently. This is normally not the case in large scale autothermal gasifiers, where instead λ, in practice, controls the process temperature during gasification (e.g., [40]). A higher λ means more oxygen to the gasifier, resulting in a higher process temperature due to the heat release from the enhanced exothermic combustion reactions. In this work, the gasification experiments were operated at three different λ setpoints (0.35, 0.43, and 0.50), and at two different process temperatures (1200 and 1300 °C). The λ setpoints were chosen based on previous experience and set within a range where high gasification efficiency was expected [40]. Temperatures of the DTF were set to match process temperatures expected from full-scale autothermal gasification of the feedstock at the given λ settings.

The fuel feeding rate was about 0.8 mL/min (0.037 kg/h) and the atomizing gas, consisting of air, was varied from 2.44 to 3.49 L/min. The added air flow through the atomizer corresponded to the amount of air needed for each specific λ setpoint. The secondary gas flow consisted of nitrogen. The total gas flow (primary and secondary) into the DTF was kept at about 11 L/min for all operational setpoints.

### 3.4. Gasification Performance Parameters

To reliably calculate key performance parameters from the gasification experiments it was important to close the mass balance during the experiments. The inputs to the mass balance calculations were mass flow rates of C, H, and O into the DTF from the feedstock and the atomization air, respectively. The output flowrates of C, H, and O could be calculated from the syngas composition (µGC and FTIR) and the syngas flowrate. As the flowrate of nitrogen to the process is known (controlled via air and nitrogen mass flow controllers (MFCs), respectively) and the nitrogen concentration was measured on the µGC, the total syngas flowrate could be calculated.

Generally, the cold gas efficiency (CGE) is used as a measure of the gasification process efficiency [39]. The CGE is generally defined as the ratio between the chemical energy in the cooled syngas and the energy input from the fuel. The CGE is normally based on the lower heating values (LHV) of the produced gas and fuel, respectively.

If the produced syngas is intended to be used for power production, for example burnt in a gas turbine, all the energetic gas components of the syngas is included in the CGE. This efficiency number is hereafter referred to as *CGE_powe_*_r_ [62]. However, when the produced gas is intended for downstream synthesis of chemicals or motor fuels, only H_2_ and CO are the important syngas species for the synthesis of, for example, methanol [63] or Fischer–Tropsch fuels [64]. This latter CGE is hereafter referred to as *CGE_chem_*. In addition to the CGE, the relation of the main gas components (H_2_, CO, and CO_2_) can be an important parameter depending on what product is desired. For example, the molar ratio of H_2_/CO is a valuable process parameter to evaluate when looking at downstream chemical synthesis [63,64].

## 4. Conclusions

The conclusions from this work are summarized as follows:

Pyrolysis of plastic waste could be an opportunity to introduce a recycled and pumpable feedstock for entrained flow gasifiers in the petrochemical industry for the production of high-quality syngas.

Pyrolysis of polypropylene in a fluidized bed reactor at 600 °C resulted in gases, liquid product, and waxy material with a melting temperature of approximately 98 °C. The liquid and wax had similar elemental compositions (C, H, N) and heating values. The liquid product consisted mainly of lighter hydrocarbons (<C_20_), whereas the wax fraction was enriched in hydrocarbons larger than C_20_.

A solid carbonaceous residue was formed during gasification of the pyrolysis liquid, mostly from low gasification temperatures and low λ-setpoints. It was concluded that the material was composed of soot with turbostratic structure.

The highest cold gas efficiencies (CGE) and H_2_/CO ratio during gasification were obtained at 1300 °C and λ = 0.35. However, low λ settings also resulted in increased yield of soot, which means that there is a balance between high efficiency and a clean syngas.

Overall, the bench scale experimental yields of CO and H_2_ in this two-step process were estimated to 0.95 and 0.06 kg per kg of polypropylene, respectively, assuming that the pyrolysis liquid and wax can be combined. These numbers were estimated based on the gasification yields at λ = 0.43 and 1300 °C, which gave the highest carbon conversion efficiency from polypropylene to CO (48 wt.%). On an energy basis, the energy content of CO and H_2_ in the produced syngas corresponded to approximately 40% of the energy content of the polypropylene raw material (based on the lower heating values, LHV). These numbers are, however, expected to be significantly improved on a larger scale where losses are proportionally smaller.

## Figures and Tables

**Table 1:** Product yields for gases, liquids, and waxes from the pyrolysis of polypropylene.

Fraction	wt.%
Pyrolytic gases	17
Organic liquids	14
Organic waxes	53
Organic product total	67
Sum of products	84

**Table 2:** Yield of individual gas components (wt.% of feedstock) during pyrolysis of polypropylene.

Gas Component	Yield (wt.%)
Methane	1.0
Ethane	1.3
Ethene	1.4
Propane	0.4
Propene	7.6
n-Butane	0.1
*trans*-2-Butane	0.1
1-Butene	0.2
i-Butene	1.3
1,3-Butadiene	0.2
n-Pentane	0.4
1-Pentene	0.2
Benzene	0.1
Other C_3+_	2.8

**Table 3:** Physicochemical properties of the pyrolysis liquid and wax.

	C	H	N	H/C	HHV	LHV	Viscosity at 40 °C	Density at 40 °C	Melting Temp.	Hydrocarbon Range			
	wt.%	wt.%	wt.%	-	MJ/kg	MJ/kg	mm^2^/s	g/cm^3^	°C	C_7_–C_20_	C_21_–C_37_	C_37+_	End Point
Liquid	83.9	13.8	<0.1	0.16	46.3	43.3	2.3	0.77	n.m.	70	22	8	C_77_
Wax	83.7	13.8	<0.1	0.16	46.2	43.2	n.m.	n.m.	98	47	27	26	C_102_

HHV = Higher heating value, LHV = Lower heating value, n.m. = not measured.

**Table 4:** Yield of gas components (mol/kg fuel) from gasification experiments at different operating conditions.

Gas Specie	Yield (mol/kg Fuel) 1200 °C			Yield (mol/kg Fuel) 1300 °C		
	λ 0.35	λ 0.43	λ 0.50	λ 0.35	λ 0.43	λ 0.50
H_2_	44.4	37.0	34.9	52.4	46.6	40.6
CO	41.6	42.4	44.4	48.3	51.2	51.1
H_2_O	17.3	23.3	28.7	15.5	21.2	27.3
CO_2_	7.9	12.8	17.6	6.2	10.1	15.2
CH_4_	2.4	2.2	2.2	1.6	1.6	1.5
C_2_H_2_	1.9	1.5	1.2	1.8	1.2	0.7
C_2_H_4_	0.13	0.09	0.07	0.12	0.08	0.05

**Table 5:** The calculated mass balances for carbon, hydrogen, and oxygen from the gasification experiments.

	Massbalance (wt.%) 1200 °C			Massbalance (wt.%) 1300 °C		
λ	C ^1^	H	O	C ^1^	H	O
0.35	80	101	104	86	105	103
0.43	87	97	103	94	106	103
0.50	96	100	102	99	107	103

^1^ Only gaseous species (CO, CO_2_, CH_4_, C_2_H_2_, and C_2_H_4_).

## Data Availability

The data presented in this study are available on request from the corresponding author.

## Sample Availability

Not available.
